# Metallic Nanoparticle Block Copoloymer Vesicles with Enhanced Optical Properties

**DOI:** 10.3390/nano1010020

**Published:** 2011-05-09

**Authors:** Juan Leonardo Martinez-Hurtado

**Affiliations:** Institute of Biotechnology, University of Cambridge, Tennis Court Rd, CB21QT, Cambridge, UK; E-Mail: juanleonardo@gmail.com; Tel.: +44-(0)-1223-767783; Fax: +44-(0)-1223-334162

**Keywords:** block copolymer, metallic nanoparticle, optical properties, encapsulation

## Abstract

The fabrication and characterization of template silver nanoshell structures and the encapsulation of gold nanoparticles using biocompatible poly(oxyethylene)-poly(butylene) diblock co-polymer vesicles is described in this work. These vesicles have a narrow diameter size distribution around 200 nm. Silver nanoparticles (*ϕ* = 1–10 nm) functionalized with decanethiol were successfully entrapped in the hydrophobic membrane and non-functionalized gold nanoparticles (*ϕ* = 3.0–5.5 nm) were encapsulated in the vesicle core. Transmission Electron Microscopy confirms the localisation of the particles; silver functionalized nanoparticles appear to thicken the vesicle membrane as shown with TEM image analysis. The enhancement of the optical properties is confirmed using transmission spectrophotometry; the 430 nm plasmon resonance peak of the silver nanoparticles was replaced by a broader extinction spectrum to beyond 700 nm (O.D. = 0.8). For a number density of 4.8 × 10^12^ mL^−1^ the scattering cross section was calculated to be 0.92 × 10^−4^ *μ*m^2^ with a scattering coefficient of 0.44 mm^−1^. The measurements indicate scattering cross section of 3.8 × 10^−5^ *μ*m^2^, attenuation coefficient of 0.18 mm^−1^ and extinction efficiency equal to 1.2 × 10^−3^. Stable and biocompatible block co-polymer vesicles can potentially be used as plasmon-resonant optical contrast agents for biomedical applications.

## 1. Introduction

### 1.1. Block-Copolymer Vesicles

Poly(oxyethylene)-poly(butylene) diblock co-polymer (PEO-PBO) is known to form stable vesicular structures in solution [1]. Polymerosomes are formed in a controlled stepwise process from a lamellar film structure to solution-filled protuberances and finally to vesicles [2]. The geometry of these structures is controlled by the length of the polymer chains and the final polymer concentration in aqueous solution [3–7]. Once the vesicles are formed, the diameter size can be controlled by mechanical extrusion of the solution through a porous membrane with defined porous size [8]. These polymer vesicles can be used as intracellular delivery systems giving more stability and circulation times in blood than the traditional systems [8,9]. Furthermore, these well-designed nanostructures can overcome biocompatibility issues [10], making them suitable for biomedical applications [11,12].

### 1.2. Contrast Agents and Metallic Nanoparticles

Metallic nanoparticles have been of particular interest because of their high surface plasmon-resonance [13]. Gold and silver nanoparticles are excellent in absorbing and scattering visible and infrared radiation [14,15]. Different sizes and shapes of nanoparticles give different absorption and scattering properties [13] which make them of great interest for biological imaging applications [16,17]. Because biological tissues disturb the photon trajectories and can be difficult to image at visible wavelengths, the use of near infrared radiation and contrast agents [18–20] is required because longer wavelength infrared radiation can penetrate tissue in greater depth.

### 1.3. Polymerosme-Metallic Nanoparticle Constructions

It has been shown that it is possible to construct templates for metallic nanoparticles by interaction of the particles and self-assembling biocompatible materials [21–25]. The possibility of combining the optical advantages offered by the nanoparticles and the controlled assembly of PEO-PBO polymerosomes is explored in this work.

## 2. Experimental Section

The diblock copolymers synthesized by sequential anionic polymerization [26] were provided by the Department of Chemistry, University of Sheffield, UK. The copolymer contains 16 units of poly(oxyethylene) and 22 units of poly(oxybutylene) [7]. Gold metallic nanoparticles (G1402, gold colloid solution 0.01% HAuCl, 3.0–5.5 nm mean particle diameter) and silver nanoparticles (673633, decanethiol functionalized silver nanoparticles, 0.1% Ag-SCH_2_(CH_2_)_8_CH_3_ in hexane, 1–10 nm particle size) were purchased from Aldrich and used upon arrival.

### 2.1. Vesicle Formation

**Figure 1.**
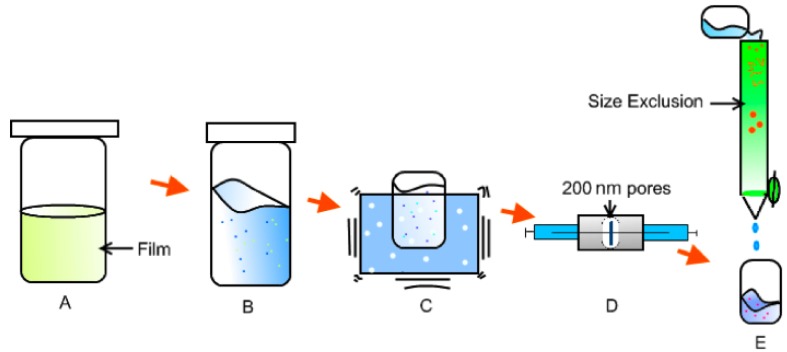
Vesicle formation and encapsulation process. **(A)** Copolymer film formation by solvent evaporation; **(B)** Disruption of the copolymer lamellar structure forming polymer aggregates in solution by stirring; **(C)** Perfusion of the particles in solution into the polymer vesicles by sonication; **(D)** Vesicle extrusion to narrow the diameter size distribution to 200 nm; **(E)** Size exclusion separation of the polymer vesicles from non-encapsulated particles and polymers through a sepharose column.

The copolymer is a viscous transparent liquid that forms very stable vesicles at the critical vesicle formation concentration [6]. In order to reach that concentration, solutions of 10 mg of polymer in 3 mL chloroform were prepared using glass vials. The chloroform promotes the separation and dispersion of the concentrated copolymer chains. Once the polymer was completely dissolved, glass vials containing this solution were left open in a vacuum oven at 50 °C overnight. This process generates a thin film on walls of the glass vial as shown in Figure 1A.

The film was then brought to the critical vesicle formation concentration of 10 mg/mL [5], with phosphate buffer, stirring for 1 h (Figure 1B). This process allows the separation of the highly packed lamellar structures forming polymer aggregates in solution [2,5]. The solution was sonicated for 10 min in a water bath sonicator (70–80 kHz, Sonicor Instruments). This promotes the vesicle formation and disrupts the polymer aggregates, thus enabling the encapsulation of the particles in solution. (Figure 1C). The formed vesicles were then homogenized in size using an extrusion apparatus (Figure 1D) LiposoFast-Basic [27]. The extrusion was performed by passing the solution through a polycarbonate membrane with a 200 nm pore size (Avestin, Inc). This process narrows the vesicle size distribution to about 200 nm in diameter [8]; analogous process have shown encapsulation efficiency of 20% using this technique [28]. The extruded solution is eluted using a size exclusion column (d = 1 cm × H = 12 cm) filled with Sepharose 4B (Sigma-Aldrich). The aliquots contained solutions of the polymerosomes separated from non-assembled polymer chains and non-encapsulated particles (Figure 1E). This step dilutes the samples to a 20% of the initial concentration.

### 2.2. Particle Encapsulation

**Figure 2.**
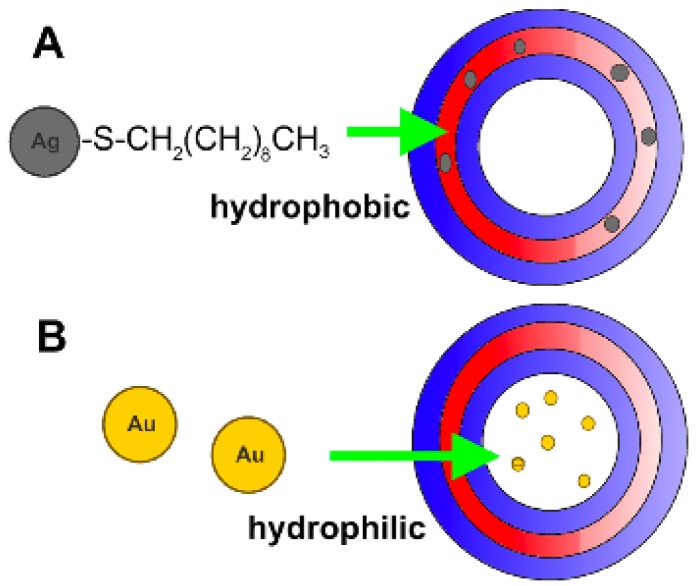
Nanoparticle encapsulation exemplification. **(A)** Hydrophobic functionalized silver particles entrapped in the membrane; and **(B)** hydrophilic gold nanoparticles encapsulated in the core. Red and blue areas depict the hydrophobic and hydrophilic domains of the block copolymer in the vesicles.

The gold nanoparticles in aqueous solution (0.01%) were incorporated in step B in Figure 1. The decanethiol functionalized silver nanoparticles in hexane (*i.e.*, 3 mL of 0.1% Ag-SCH_2_(CH_2_)_8_CH_3_ sol.) were added before the film formation in step A in Figure 1, together with the chloroform for an estimated final concentration in the vesicles of 0.06%. The hydrophilic-hydrophobic nature of the nanoparticles permits to allocate the particles in the membrane (Figure 2A) or the vesicle core (Figure 2B).

### 2.3. Electron Microscopy Imaging

Samples of encapsulated gold and silver were mounted into carbon coated copper grids with no metal enhancement or staining. The grids were treated with uranyl formate solution 2% w/w and dehydrated with ethanol. The images were recorded in the transmission electron microscope (Phillips CM100 TEM) with a Gatan CCD camera and analysed with a TEM image processing software DigitalMicrograph v3.6.5 (Gatan Inc).

### 2.4. Transmission Spectrophotometry

The transmission spectrophotometry was performed with a double beam, double monochromator, Lambda 900 Perkin-Elmer UV-Vis-IR spectrophotometer. The capture speed was set to 600 nm/min with a beam slit of 1 nm, using 1 cm quartz cuvettes with burnished sides.

### 2.5. Scattering Calculations

The scattering efficiency of light passing through a homogeneous medium with spherical particles in solution can be calculated using the Mie solution for the Maxwell's equations. For this solution, it is required to specify the refractive index of the medium, the refractive index of the particles, the wavelength of the incident light, the particle diameter and the particle concentration. We calculated these parameters considering the membrane thickness *t*, the vesicle diameter *D* and the values reported in Table 1.

With a mean diameter of 200 nm the volume occupied by the membrane *V_m_* is calculated as

(1) $$V_{m}=\frac{3}{4}\pi \;{\left(\frac{D}{2}\right)}^{3}-\frac{3}{4}\pi \;{\left(\frac{D-2t}{2}\right)}^{3}$$

with this value it is possible to calculate the polymer units per vesicle *P_υ_*

(2) $$P_{\upsilon }=\frac{V_{m}}{\upsilon_{m}}$$

the mass of the polymer in the per vesicle *m_υ_*

(3) $$m_{\upsilon }=\frac{P_{\upsilon }xMW}{N_{A}}$$

and the vesicles concentration *η*

(4) $$\eta =\frac{C}{m_{\upsilon }}$$

The vesicles concentration or number density *η* was calculated to be 2.412 × 10^16^ L^−1^. This value is affected by a dilution of 20% of the initial concentration (*i.e.*, *η* = 4.8 × 10^12^ mL^−1^). Using Mie scatter calculation software [29] it is possible to calculate the scattering properties of the particles using Mie theory. Calculations were done using *λ* = 700 nm and refractive index of *n_m_* = 1.33 for the medium and *n_Ag_* = 1.54 for the Ag particles as required for this calculation, reported elsewhere [30–33] for Ag metal particles. The output gives the scattering cross section, *σ_t_*. This parameter divided by the cross-sectional area gives the scattering efficiency parameter *Q_t_*

(5) $$Q_{t}=\frac{\sigma_{t}}{\pi r^{2}}$$

The total attenuation coefficient is given by the scattering cross section multiplied by the number density of the scatterers.

(6) $$\mu_{t}=\sigma_{t}\eta$$

The script gives values for the total attenuation coefficient and scattering cross section of *μ_t_* = 0.44 mm^−1^ and *σ_t_* = 0.92 × 10^−4^*μ*m^2^ respectively. Assuming that the silver particles are allocated in the membrane acting as a template for the silver nanoshells formation.

## 3. Results and Discussion

### 3.1. Transmission Electron Microscopy

**Figure 3.**
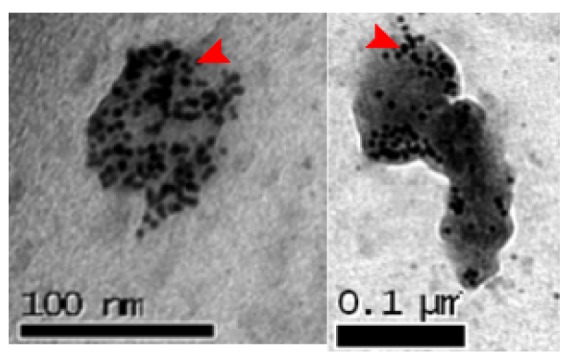
TEM images of encapsulated non-functionalized gold nanoparticles. Red arrows point at spherical gold particles.

It was found that the non-functionalized gold nanoparticles were encapsulated inside the vesicles without forming any uniform structure. Figure 3 shows the enhanced contrast of the nanoparticles encapsulated in the polymer vesicles. As the vesicles are made of a soft polymeric material, the electron beam of the electron microscope progressively damages the vesicle membrane and deforms their original shape, as it can be seen in the figure [34]. However, the contrast between the vesicle material, the nanoparticles and the surrounding media can still be observed.

**Figure 4.**
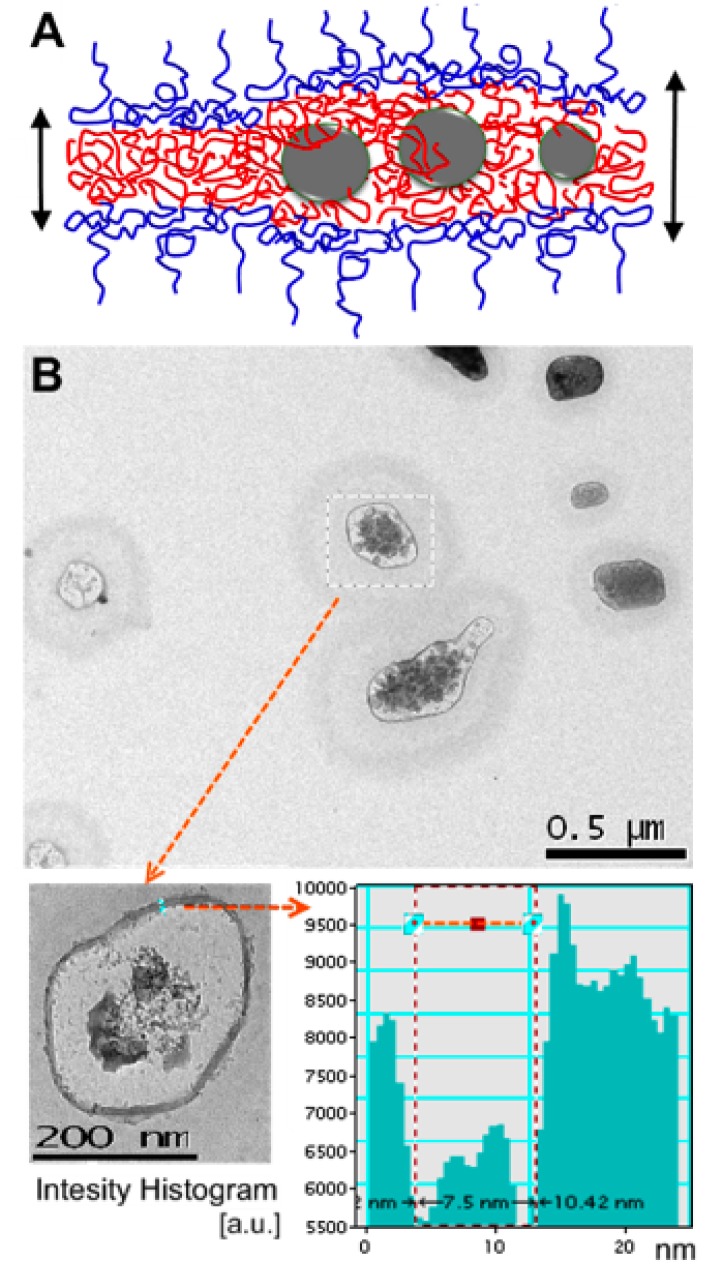
**(A)** Schematic representation of the nanoparticles thickening the membrane; The normal thickness of the PEO-PBO vesicle membranes is 2.4 nm [7]; **(B)** TEM images of vesicles showing the membrane thickening up to 7.5 nm and enhanced contrast for TEM, together with intensity histogram analysis (DigitalMicrograph v3.6.5, Gatan Inc).

Silver nanoparticles coated with hydrophobic chains would interact with the hydrophobic domain of the polymer during the vesicle self-assembly. Therefore, functionalized silver nanoparticles were expected to be found entrapped in the membrane. Figure 4A shows a schematic representation of the membrane entrapped particles. It was also found that the inclusion of silver nanoparticles in the membrane increased the membrane thickness from *t* = 2.4 nm to *t* ≈ 6.1 ± 1.3 nm (see Table 1), indicating the particle entrapment. The particles contribute to not only the membrane thickening but also the enhanced contrast and optical properties. The polymers are soft materials that lack sufficient contrast for a TEM to be seen. Although PEO-PBO have proven to have high stability for imaging the membranes, the soft spheres can be damaged at high magnifications by the electron beam [7]. Here, however, the membranes can still be seen with enhanced contrast as observed in Figure 4B. These results also suggest that only small silver particles from the solution (*ϕ* = 1–5 nm) were encapsulated and larger particles were eliminated during the encapsulation process. The membranes collapsed when the electron beam is focused at the vesicles because, since the Ag particles are localized in the membrane, it is difficult to determine the presence of these particles within the core.

### 3.2. Transmission Spectrophotometry

**Figure 5.**
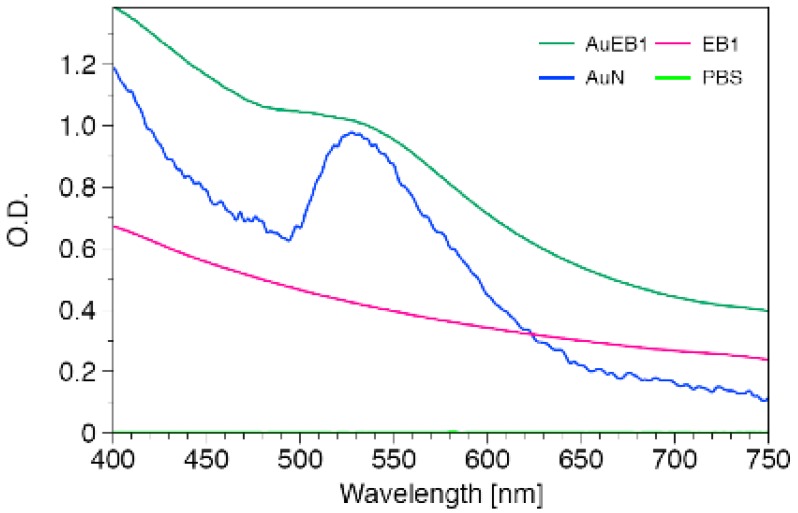
Optical spectrum of encapsulated gold nanoparticles (AuEB1), gold nanoparticles solution 0.04 mg/mL (AuN), PEO-PBO block copolymer vesicles only (EB1), and solution buffer (PBS).

In order to corroborate the actual enhancement of the optical properties of the silver nanoshells, it is necessary to compare the optical transmission spectrum for the different constructions. It has been shown elsewhere that the configuration of nanoparticles in space can lead to changes in the surface plasmon resonance of the engineered conglomeration of particles [35]. Figure 5 shows the comparison of the absorption spectrum of the gold nanoparticles, the encapsulated gold nanoparticles and the PEO-PBO vesicles. The solutions containing gold nanoparticles showed a small peak where the surface plasmon resonance of gold appears. However, non-enhancement of the optical properties was appreciated. As the particles are randomly distributed in the core of the vesicles, the optical properties remain the same as in solution; therefore, the optical absorption is only modified by the scatter of the cloudy polymer solutions. Therefore, encapsulation of gold nanoparticles in the core did not produce vesicles with enhanced optical properties.

**Figure 6.**
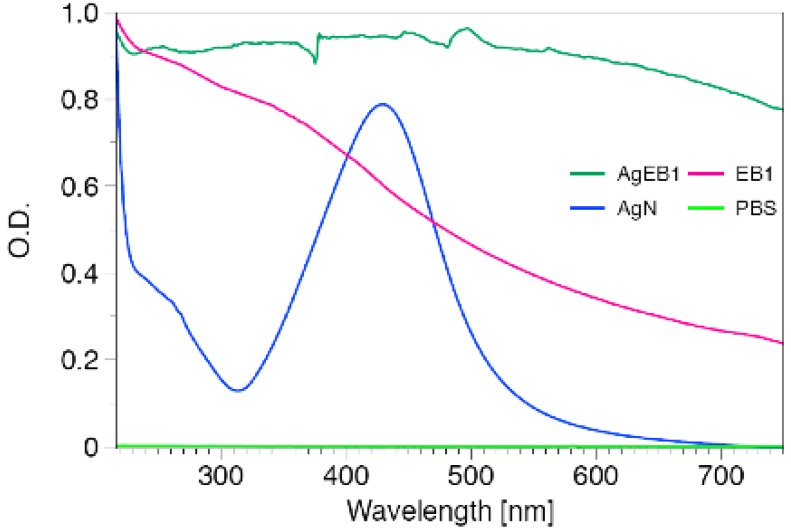
Optical spectrum of membrane entrapped functionalized silver nanoparticles (AgEB1), silver nanoparticles solution 0.6 mg/mL (AgN), PEO-PBO block copolymer vesicles (EB1), and solution buffer (PBS).

Figure 6 shows the spectrum comparison for silver nanoparticles, membrane entrapped silver nanoparticles and PEO-PBO vesicles. The functionalized silver nanoparticles encapsulated in the membranes for a shell that could potentially lead to surface plasmon resonance variations [35]. In the experiment described here, the entrapment of silver nanoparticles in the vesicle membranes caused a change in the optical properties of the vesicles. The surface plasmon peak expected for silver appears for the silver nanoparticle solutions but it seems to be modified for the vesicle containing silver nanoparticles in the shell. This construction shows that encapsulated silver nanoparticles have a broader extinction spectrum to beyond 700 nm with an optical absorption greater than 0.8 O.D., as shown in Figure 6; in fact, the enhancement occurs for *λ* = 210–700 nm. Silver nanoparticles do not usually absorb at those wavelengths when in a colloidal solution; after being assembled in the vesicle membranes the combined optical properties are enhanced. Some applications that require absorption in the near infrared for non-invasive diagnosis could potentially use encapsulated contrast enhancers like the one described here.

### 3.3. Optical Densities and Attenuation Coefficient

It is possible to relate the spectrophotometry measurements of optical density to the total attenuation coefficient from Equation (6) using the Lambert-Beer law

(7) $$O.D.=-\log_{10}\left(\frac{I}{I_{0}}\right)$$

(8) $$\frac{I}{I_{0}}=\text{exp}(-\eta \sigma_{t}l)$$

where *I*_0_ is the intensity of the incident beam, *I* the intensity of the transmitted light and *l* the path length; giving *μ_t_* = 0.23 O.D. in mm^−1^. For *λ* = 700 nm, the total attenuation coefficient *μ_t_* is 0.18 mm^−1^. This equates an extinction cross section *σ_t_* of 3.8 × 10^−5^ and extinction efficiency *Q_t_* = 1.2 × 10^−3^. Knowing the extinction efficiency of the material is important for its use in certain biomedical applications in which contrast agents are required to differentiate from normal tissue scatter or absorption, so that the material can be compared and used if suitable. For example, some studies report that optical tomography scanners require scatter coefficients *μ_s_* above 0.5 cm^−1^, other studies suggest *μ_s_* = 2–6 mm^−1^ (*μ_t_* = *μ_a_* + *μ_s_*, *μ_a_* absorption coefficient) [36–38]. Certain parameters can be improved for better extinction efficiency (*i.e.*, concentration of the particles, diameter size, *etc.*). Using biocompatible nanostructures with controllable size and shape as templates for silver nanoshells gives advantages for bioimaging techniques that require near infrared scatter.

## 4. Conclusions

Metallic nanoparticles were successfully incorporated into self-assembled PEO-PBO diblock copolymer vesicles. Non-functionalized gold nanoparticles were encapsulated in the vesicle core whereas silver decanethiol functionalized nanoparticles were entrapped in the membrane. The silver nanoparticles were shown to thicken the vesicle membranes, giving enhanced optical properties. Since the PEO-PBO vesicles are biocompatible, the metallic nanoparticle templates can potentially be used as contrast agents in the near infrared for bioimaging applications.

## Figures and Tables

**Table 1.** Data for PEO-PBO polymerosomes [7].

Area per molecule	Volume per molecule	Membrane thickness
*a_m_* = 1.130 nm^2^	*υ_m_* = 2.712 nm^3^	*t* = 2.4 nm

Molecular weight	Avogadro's number	Polymer concentration
MW = 2300	*N_A_* = 6.022 × 10^23^ mol^−1^	C = 10 gL^−1^

## References

[1] Discher D., Eisenberg A. (2002). Polymer vesicles. Science.

[2] Battaglia G., Ryan A. (2006). Pathways of polymeric vesicle formation. J. Phys. Chem. B.

[3] Battaglia G., Ryan A. (2006). Neuron-like tubular membranes made of diblock copolymer amphiphiles. Angew. Chem. Int. Ed..

[4] Smart T. (2008). Block copolymer nanostructures. Nanotoday.

[5] Battaglia G., Ryan A. (2005). The evolution of vesicles from bulk lamellar gels. Nat. Mater..

[6] Battaglia G., Ryan A. (2006). Effect of amphiphile size on the transformation from a lyotropic gel to a vesicular dispersion. Macromolecules.

[7] Battaglia G., Ryan A. (2005). Bilayers and interdigitation in block copolymer vesicles. J. Am. Chem. Soc..

[8] Lomas H. (2008). Non-cytotoxic polymer vesicles for rapid and efficient intracellular delivery. Faraday Discuss..

[9] Photos P.J., Bacakovaa L., Dischera B., Batesb F.S., Discher D.E. (2003). Polymer vesicles in vivo: Correlations with PEG molecular weight. J. Control. Release.

[10] Hearnden V. (2009). Diffusion studies of nanometer polymersomes across tissue engineered human oral mucosa. Pharm. Res..

[11] Discher D. (2007). Emerging applications of polymersomes in delivery: From molecular dynamics to shrinkage of tumors. Prog. Polym. Sci..

[12] Hughes G.A. (2005). Nanostructure-mediated drug delivery. Nanomedicine.

[13] Jain P., Lee K., El-Sayed I. (2006). Calculated absorption and scattering properties of gold nanoparticles of different size, shape and composition: Applications in biological imaging and biomedicine. J. Phys. Chem..

[14] El-Sayed I., Huang X., El-Sayed M. (2005). Surface plasmon resonance scattering and adsorption of anti-EGFR antibody conjugated gold nanoparticles in cancer diagnostics: Applications in oral cancer. Nano Lett..

[15] Rosi N., Mirkin C.A. (2005). Nanostructures in biodiagnostics. Chem. Rev..

[16] Ghoroghchian P. (2005). Near-infrared-emissive polymerosomes: Self-assembled soft matter for in vivo optical imaging. Proc. Nat. Acad. Sci. USA.

[17] Lee T. (2003). Engineered microsphere contrast agents for optical coherence tomography. Optic. Lett..

[18] Izatt J., Kulkarni M. (1996). Optical coherence tomography and microscopy in gastrointestinal tissues. IEEE J. Sel. Top. Quantum Electr..

[19] Masato O., Masamitsu H. (2003). Ultra-high resolution optical coherence tomography (OCT) using a halogen lamp as the light source. Opt. Rev..

[20] Boppart J., Hoying J., Sullivan C. (2005). Optical probes and techniques for molecular contrast agents for spectroscopic optical coherence tomography. Opt. Lett..

[21] Yu M., Wang H., Zhou X. (2007). One template synthesis of raspberry-like hierarchical siliceous hollow spheres. J. Am. Chem. Soc..

[22] Wang R. (2006). Self-assembled gold nanoshells on biodegradable chitosan fibers. Biomacromolecules.

[23] Li Y. (2007). *In Situ* formation of Gold-“Decorated” vesicles from a RAFT-synthesized, thermally responsive block copolymer. Macromolecules.

[24] Troutman T., Barton J., Romanowski M. (2008). Biodegradable plasmon resonant nanoshells. Adv. Mater..

[25] Yuan J.J. (2006). Facile synthesis of highly biocompatible poly(2-(methacryloyloxy)ethyl phosphorylchline)-coated gold nanoparticles in aqueous solution. Langmuir.

[26] Booth C., Yu G., Nace V., Lindman B., Alexandridis P. (2000). Self-assembly in simple and complex systems. Amphiphilic Block Copolymers.

[27] MacDonald R.C., MacDonald R.I., Menco B.P., Takeshita K., Subbarao N.K., Hu L.R. (1991). Small-volume extrusion apparatus for preparation of large unilamellar vesicles. Biochim. Biophys. Acta.

[28] Lomas H. (2007). Biomimetic pH sensitive polymersomes for efficient DNA encapsulation and delivery. Adv. Mater..

[29] Prahl S. (2007). Mie Scattering Calculator.

[30] Malynych S., Chumanov G. (2006). Coupled planar silver nanoparticle arrays as refractive index sensors. J. Opt. A-Pure Appl. Opt..

[31] Johnson P., Christy R. (1972). Optical constants of the nobel metals. Phys. Rev. B.

[32] Scaffardi L., Tocho J. (2006). Size dependence of refractive index of gold nanoparticles. Nanotechnology.

[33] Curry A., Nusz G., Chilkoti A., Wax A. (2005). Substrate effect on refractive index dependence of plasmon resonance for individual silver nanoparticles observed using darkfield micro-spectroscopy. Opt. Express.

[34] Chen J.T., Thomas E.L. (1996). The use of force modulation microscopy to investigate block copolymer morphology. J. Mater. Sci..

[35] Oldenburg S.J., Averitt R.D., Westcott S.L., Halas N.J. (1998). Nanoengineering of optical resonance. Chem. Phys. Lett..

[36] Agrawal A., Pfefer T.J., Huang S., Lin A.W.H., Lee M.-H., Drezek R.A., Barton J.K. (2006). Quantitative evaluation of optical coherence tomography signal enhancement with gold nanoshells. J. Biomed. Opt..

[37] Lu Q., Gan X., Gu M., Luo Q. (2004). Monte carlo modeling of optical coherence tomography imaging through turbid media. Appl. Opt..

[38] Faber D., van der Meer F., Aalders M., van Leeuwen T. (2004). Quantitative measurement of attenuation coefficients of weakly scattering media using optical coherence tomography. Opt. Express.

